# Two myeloid leukemia cases with rare *FLT3* fusions

**DOI:** 10.1101/mcs.a003079

**Published:** 2018-12

**Authors:** Haijiao Zhang, Aleksandra Paliga, Evie Hobbs, Stephen Moore, Susan Olson, Nicola Long, Kim-Hien T. Dao, Jeffrey W. Tyner

**Affiliations:** 1 Department of Cell, Developmental and Cancer Biology, Oregon Health and Science University, Knight Cancer Institute, Portland, Oregon 97239, USA;; 2 Division of Hematology and Medical Oncology, Oregon Health and Science University, Knight Cancer Institute, Portland, Oregon 97239, USA;; 3 Department of Molecular and Medical Genetics, Oregon Health and Science University, Knight Cancer Institute, Portland, Oregon 97239, USA

**Keywords:** chronic myelomonocytic leukemia, transient myeloproliferative syndrome

## Abstract

Genetic rearrangements involving *FLT3* are rare and only recently have been detected in myeloid/lymphoid neoplasms associated with eosinophilia (MLN-eos) and chronic myeloproliferative disorders. Here we report two cases with *FLT3* fusions in patients demonstrating mixed features of myelodysplastic/myeloproliferative neoplasms. In the first case, *FLT3* was fused with a new fusion partner *MYO18A* in a patient with marrow features most consistent with atypical chronic myeloid leukemia; the second case involving *ETV6*-*FLT3* fusion was observed in a case with bone marrow features most consistent with chronic myelomonocytic leukemia. Notably, we observed that samples from both patients demonstrated FLT3 inhibitor (quizartinib and sorafenib) sensitivity in ex vivo drug screening assay.

## INTRODUCTION

Atypical chronic myeloid leukemia (aCML) and chronic myelomonocytic leukemia (CMML) are rare myeloid clonal stem cell disorders under the classification of myelodysplastic/myeloproliferative neoplasms (MDS/MPN) with an inherent tendency to transform into acute myeloid leukemia (AML) (Wandt et al. 2010). The FMS-like tyrosine kinase (*FLT3*) gene is located on Chromosome 13q12 and likely drives myeloproliferation through up-regulation of RAS and PI3K pathways. Genetic rearrangements involving *FLT3* are rare and only recently have been detected in myeloid/lymphoid neoplasms associated with eosinophilia (MLN-eos) and chronic myeloproliferative disorders (Stirewalt and Radich 2003). To date, 10 clinical cases and four *FLT3* fusion partners have been identified: *ETV6*, *SPTBN1*, *GOLGB1*, and *TRIP11*, with *ETV6* being the most common reported partner (six cases) (Vu et al. 2006; Grand et al. 2007; Tzankov et al. 2008; Walz et al. 2011; Chonabayashi et al. 2014; Falchi et al. 2014; Hosseini et al. 2014; Chung et al. 2017; Troadec et al. 2017). In all reported cases in which chimeric transcripts were cloned, the amino-terminal portion of the predicted fusion protein was composed of a partner gene with a helix–loop–helix (HLH) or coiled-coil motif, which would promote dimerization and induce constitutive activation of *FLT3* tyrosine kinase (TK) in the carboxy-terminal portion, leading to cellular transformation. Here we report two cases with *FLT3* fusions in patients demonstrating mixed features of MPN/MDS.

## RESULTS

**Figure 1.**
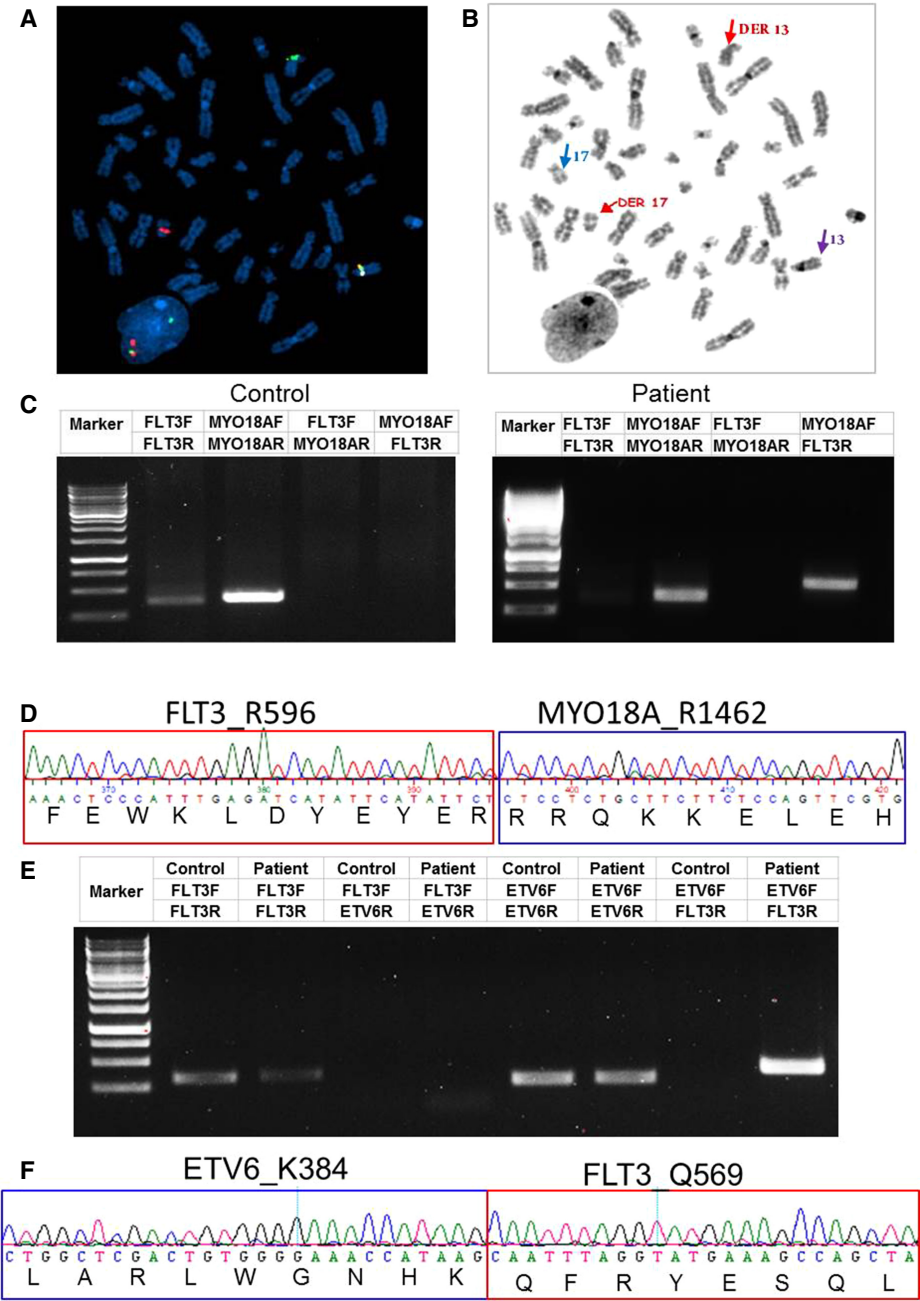
Identification of *FLT3* fusions. (*A*) FISH analysis on interphase and available metaphase cells was performed with an Agilent *FLT3* (13q12.2) break-apart probe. The FISH image shows that the distal *FLT3* signal (red; including at least exons 1–9, based on probe build) hybridized to Chromosome 17 near the centromere; the proximal *FLT3* signal (green; including at least exons 20–24, based on probe build) was retained on Chromosome 13, near the centromere, band 13q12.2. (*B*) The parallel DAPI-banded image, with the der(13), der(17), and normal homologs identified. Blue arrow: normal Chromosome 17; purple arrow: normal Chromosome 13. (*C*) PCR validation of the *MYO18A*-*FLT3* fusion transcript. A multiplex RT-PCR was designed to detect *MYO18A*-*FLT3* product with mRNA derived from Case 1's peripheral blood mononuclear cells (PBMCs). A leukemia patient without fusions was used as control. (*D*) Sequencing of the junction PCR product revealed an in-frame fusion between *MYO18AR1462* and *FLT3R596*. (*E*) Identification of the *ETV6*-*FLT3* fusion transcript. A multiplex RT-PCR was designed to detect *ETV6*-*FLT3* product with mRNA derived from Case 2's PBMC. A leukemia patient without fusions was used as a control. (*F*) Sequencing of the junction RT-PCR product revealed an in-frame fusion between *ETV6* K384 and *FLT3* Q569.

The first case is a 47-yr-old man who presented with leukocytosis (96 × 10^9^/L white blood cells [WBCs] and 48% neutrophils) and moderate splenomegaly. The bone marrow (BM) biopsy showed a hypercellular (100%) marrow with left-shifted granulocytic hyperplasia and mild to moderate granulocytic dysplasia as well as atypical megakaryocytic dysplasia. The marrow findings were interpreted as being suggestive of an atypical CML or, less likely, a profibrotic stage myelofibrosis. A small CD20-positive λ restricted plasma cell population (5%) was also identified and interpreted as consistent with monoclonal gammopathy of undetermined significance (MGUS). Fluorescence in situ hybridization (FISH) and real-time RT-PCR on the BM were all negative for BCR/ABL1, JAK2, and PDGFR rearrangements. An AML/MDS-targeted gene panel (Supplemental Table S1) was negative for mutations of common hematological malignancy-associated genes including *JAK2*, *CALR*, *DNMT3A*, *NPM1*, *FLT3*, etc. G-banded karyotype analysis showed a 46,XY,t(13;17)(q12;q12) balanced translocation in all 20 metaphase cells examined (Fig. 1A). As *FLT3* is one of the genes located at 13q12, we assessed the status of the *FLT3* locus with *FLT3* break-apart probes, which revealed a *FLT3* split signal pattern in 165/200 cells. On available metaphase cells, the distal *FLT3* signal (including at least exons 1–9, based on probe build) hybridized to Chromosome 17 near the centromere, and the proximal *FLT3* signal (including at least exons 20–24, based on probe build) was retained on Chromosome 13, near the centromere (Fig. 1B). To identify the fusion partner, we performed retrospective RNA sequencing. Consistent with G-banding and FISH analysis, two chromosomal translocations between *MYO18A* on Chromosomes 17 and *FLT3* on Chromosome 13 were identified (Table 1). RT-PCR of cDNA using transcript-specific primers covering one of the most abundant fusion indeed detected this *MYO18A*-*FLT3* fusion transcript, whereas the reciprocal FLT3-MYO18A fusion was not found. Sequence analysis of the RT-PCR product confirmed the fusion breakpoint encoding R1462 of *MYO18A* and R596 of *FLT3* (Fig. 1C,D).

**Table 1.** *FLT3* fusions detected by RNA-seq

SampleID	Left gene	Left chromosome	Left position	Right gene	Right chromosome	Right position	Number of reads spanning fusion	Number of spanning mate pairs	Number of mate pairs one end only spanning	StrandInfo	Left flanking sequence	Right flanking sequence
13-00656	*MYO18A*	17	27423335	*FLT3*	13	28608319	8	4	6	rr	ACCGTGCCACTTCCATCTC ATTATGGTAATGATACA AAGGAGGGGGGTGG	TACAGGTGACCGGCTCCT CAGATAATGAGTACTTCT ACGTTGATTTCAGA
13-00656	*MYO18A*	17	27423778	*FLT3*	13	28608270	53	4	42	rr	CTGGAGGGCCAGCAGG TCCGCAACCACGAACT GGAGAAGAAGCAGAGGAG	AGAATATGAATATGATCTC AAATGGGAGTTTCCAAG AGAAAATTTAGAGT
15-00557	*MYO18A*	17	27423335	*FLT3*	13	28608319	11	2	10	rr	ACCGTGCCACTTCCATCTC ATTATGGTAATGATACAAA GGAGGGGGGTGG	TACAGGTGACCGGCTCCT CAGATAATGAGTACTTC TACGTTGATTTCAGA
15-00557	*MYO18A*	17	27423778	*FLT3*	13	28608270	20	2	17	rr	CTGGAGGGCCAGCAGGTCC GCAACCACGAACTGGAG AAGAAGCAGAGGAG	AGAATATGAATATGATCTC AAATGGGAGTTTCCAA GAGAAAATTTAGAGT
14-00126	*ETV6*	12	12037520	*FLT3*	13	28608350	133	8	85	fr	TCCGGATAGTGGATCCCAACG GACTGGCTCGACTGTGGGG AAACCATAAG	CAATTTAGGTATGAAAGC CAGCTACAGATGGTAC AGGTGACCGGCTCCTC

The patient was placed on hydroxyurea 500–1000 mg daily for nearly 5 mo, which resulted in normalization of blood counts and disappearance of the t(13;17)(q12;q12) in blood cells by FISH analysis indicating he had obtained a complete remission at the hematologic and cytogenetic/FISH level. He stopped taking hydroxyurea after these results were obtained. This represented a highly unusual clinical response to hydroxyurea (a ribonucleotide reductase inhibitor) and suggested the possibility of synthetic lethality. Thereafter, he was on a close observation/monitoring plan. However, he developed progressive leukocytosis after ∼1 yr of stopping hydroxyurea. The repeat BM biopsy was consistent with a chronic phase MPN phenotype. Cytogenetic/FISH analysis demonstrated recurrence of the t(13;17) (q12;q12) with additional new clonal aberrations. No common leukemia driver mutations were found using the same gene panel (Supplemental Table S1). He was on ruxolitinib, a JAK1/2 inhibitor, as part of a clinical trial but this did not provide WBC control. Less than 2 mo later, he progressed with the development of stridor while allogeneic stem cell transplantation planning was underway. Workup, including CT scan, revealed airway stenosis with subglottic edema and a lesion in the right base of the tongue. A PET-CT scan showed increased FDG uptake throughout the bones and axillary and neck nodes bilaterally. Biopsy of cervical neck node showed a mononuclear cell infiltration with 2% blasts as well as diffuse immature monocytes and dysplastic myeloid cells consistent with the extramedullary manifestation of AML. The patient received radiation to his neck and subsequently underwent allogeneic stem cell transplantation from an unrelated donor. His posttransplant course was complicated by relapsed disease, liver graft versus host disease (GVHD), polymicrobial bacteremia, and aspiration pneumonia resulting in death a year after transplant.

The second case is that of a 49-yr-old male who presented with lymphadenopathy, leukocytosis (25.84 × 10^9^/l WBC), and thrombocytopenia (62 × 10^9^/l). There was an absolute neutrophilia, eosinophilia, basophilia, and monocytosis. The BM biopsy demonstrated a hypercellular marrow with trilineage hematopoiesis and an increased M:E ratio of 5.3:1. Monoblasts/promonocytes were increased and accounted for 6% of cells. There was myeloid dysplasia (hypogranular forms), including slightly increased numbers of dysplastic eosinophils. Megakaryocytes were decreased in number with increased monolobate forms. Overall, the BM findings were interpreted as most in keeping with a diagnosis of CMML-1. A concurrent lymph node biopsy demonstrated necrosis and surrounding CD43- and CD33-positive myeloid cells that were highly suggestive of the involvement of the lymph node by myeloid sarcoma (unfortunately flow cytometry was not performed). Chromosome analysis showed 46,XY,t(12;13)(p13;q12) in 20 of 20 metaphases. We performed retrospective RNA sequencing to identify gene fusion transcripts, which uncovered an *ETV6*-*FLT3* fusion. RT-PCR from patient cDNA confirmed the presence of an in-frame fusion between *ETV6* exon 6 (encoding K384) and *FLT3* exon 14 (encoding Q569) (Fig. 1E,F).

The patient was treated with induction chemotherapy with idarubicin/cytarabine in a clinical trial. He had disease relapse and underwent re-induction with cladribine, cytarabine, and filgrastim with mitoxantrone (CLAG-M) followed by unrelated donor stem cell transplantation and remained in remission afterward (>43 mo), although his transplantation was complicated by acute and chronic GVHD.

## DISCUSSION

In accordance with previously reported *FLT3* fusions, the *FLT3* breakpoints of these two cases were all located within exon 14 just upstream of the coding region for the TK domain. In Case 1, we identified *MYO18A* as a new partner of *FLT3*. *MYO18A* (myosin XVIIIA, KIAA0216, MysPDZ) is a member of the myosin superfamily and is widely expressed in the body. In hematological malignancies, *MYO18A* has been found as fusions with FGFR1, PDGFRB, and MLL in other hematopoietic malignancies (Walz et al. 2005; Ussowicz et al. 2012; Sheng et al. 2017). In all these cases, including the current one, all the predicted coiled-coil domains of normal *MYO18A* are retained in the fusion. The breakpoint of *ETV6* in Case 2 located in exon 6, which is also the common breakpoints region of *ETV6* and the fusion protein, therefore remained the HLH domain of *ETV6*. As shown for other TK fusion proteins, it is possible that the coiled-coil domain of *MYO18A* and the HLH domain of *ETV6* results in dimerization or oligomerization of the fusion proteins with consequent constitutive activation of the *FLT3* TK domain (Krause and Van Etten 2005).

**Figure 2.**
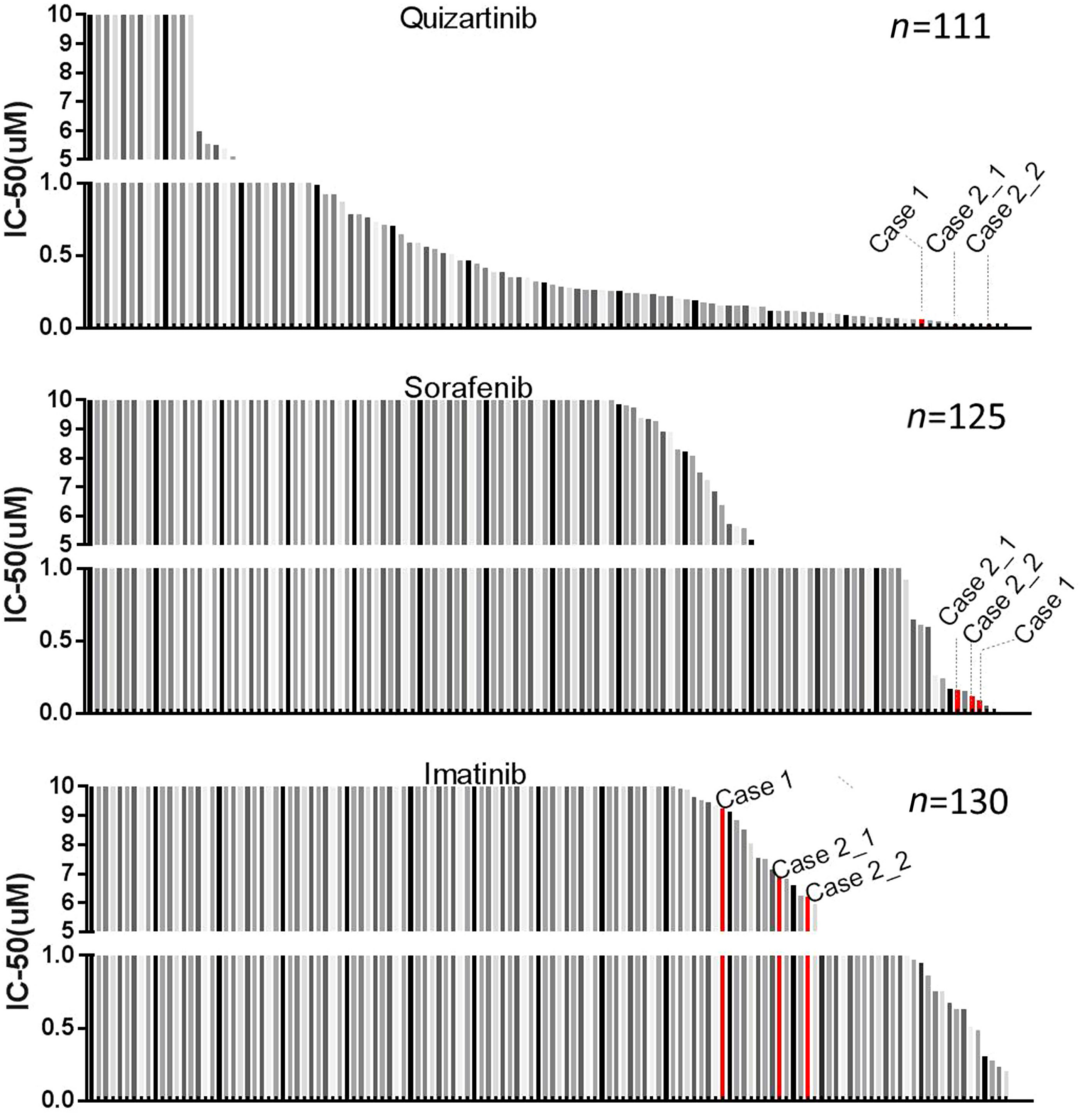
Samples with *FLT3* fusions demonstrate sensitivity to FLT3 inhibitors. PBMCs from a cohort of patients diagnosed with chronic myeloproliferative disease without BCR-ABL fusion were plated with graded concentrations of quizartinib, sorafenib, and imatinib (control) for 72 h, and cell viability was determined by a MTS assay. The graph depicts IC_50_ of a specific drug: quizartinib, sorafenib, and imatinib. Case 2_2 was harvested 1 mo after Case 2_1.

We have also performed an ex vivo drug sensitivity screening assay to evaluate the sensitivity of cells from each patient to a panel of small molecule inhibitors. We observed that samples from both patients demonstrated FLT3 inhibitor (quizartinib and sorafenib) sensitivity, but were not sensitive to a control drug (imatinib) (Fig. 2 and Supplemental Tables 3–5). This is in concordance with previous studies showing that *FLT3* fusion transformed cells and clinical cases with *FLT3* fusions are sensitive to FLT3 inhibitors (Grand et al. 2007; Falchi et al. 2014; Troadec et al. 2017).

Both cases in the current study exhibit similar clinical features: leukocytosis, hypercellular marrows with myeloid dysplasia, and extramedullary involvement. All reported cases with *FLT3* fusions in the literature demonstrate poor response and/or early relapse after conventional chemotherapy (Chung et al. 2017). Based on the in vitro results here and several clinical cases reported, FLT3 inhibitors may provide transient disease control to allow more definitive therapies such as a FLT3 inhibitor in combination with multiagent chemotherapy to improve responses, stem cell transplantation, and/or a clinical trial for patients with *FLT3* genomic alterations.

In summary, including the two cases in the current study, chromosome rearrangements involving *FLT3* exhibit some common clinical features, similar translocation arrangement, and high sensitivity (at least in vitro) to FLT3 inhibitors. This report highlights the importance of *FLT3* gene rearrangements as a diagnostic entity and the potential role of FLT3 inhibitors in these cases.

## METHODS

### Cytogenetics and Fluorescence In Situ Hybridization

GTW-banded karyotype analysis was performed as a standard clinical protocol and described according to the International System for Human Cytogenetic Nomenclature (Arsham and Shaffer 2017). FISH techniques were performed as standard clinical protocol. Briefly, for the 13;17 translocation, interphase nuclei were probed using a *FLT3* (13q12.2) break-apart probe (Agilent), comprised of flanking probes, one of which covers ∼300 kb of *FLT3*, at exons 20–24, and another one that covers ∼300 kb of *FLT3*, at exons 1–9. Samples were analyzed under an Olympus BX53 photoscope, and representative photographs were taken using GenASIs software from Applied Spectral Imaging. A minimum of 100 nuclei were scored and cases were considered positive when >15% of cells displayed split signals.

### RNA-seq Fusion Detection

RNA-seq was performed as previously described (Zhang et al. 2017). Briefly, samples were sequenced using the Agilent SureSelect Strand-Specific RNA Library Preparation Kit on the Bravo robot (Agilent) and sequenced on the HiSeq 2500 using a 100-cycle paired-end protocol. Gene assignments were based on the Ensembl build 75 gene models on GRCh37. Gene fusion data were generated using the TopHat-Fusion (v2.0.14) program using default parameters (Kim and Salzberg 2011).

### Sanger Sequencing

Sanger sequencing was performed on the RNA samples to verify mutations identified by RNA-seq. Briefly, mutations were confirmed by PCR amplification using the following primers: *FLT3* forward: 5′-CAATTCCCTTGGCACATCTT-3′; *FLT3* reverse: 5′-TTGCGTTCATCACTTTTCCA-3′; *FLT3* reverse: 5′-GCAACCTGGATTGAGACTCC-3′; *ETV6* forward: 5′-CATGCCCATTGGGAGAATAG-3′; *ETV6* reverse: 5′-TCCTGGCTCCTTCCTGATAA-3′; *MYO18A* forward: 5′-GAACAAGAGGCAGCTGGAAC-3′; and *MYO18A* reverse: 5′-GAACCCTGCAATGTCCATGT-3′. PCR products were purified using Amicon Ultra Centrifugal Filters (#UFC503096, Millipore) and sequenced with the same primers.

### Small Molecule Inhibitor Screening Assay

PBMCs from a cohort of patients diagnosed with chronic myeloproliferative disease without BCR-ABL fusion were plated with graded concentrations of quizartinib, sorafenib, and imatinib (control) for 72 h, and cell viability was determined by methanethiosulfonate (MTS) assay as previously described (Tyner et al. 2013), Briefly, cell viability was measured using a MTS-based assay (CellTiter96 Aqueous One Solution, Promega), and read at 490 nm after 1–24 h using a BioTek Synergy 2 plate reader (BioTek), Cell viability was determined by comparing the absorbance of drug-treated cells to that of untreated controls set at 100%, IC_50_ values were calculated by a regression curve fit analysis using GraphPad Prism software, and all drugs were obtained from commercial vendors.

## ADDITIONAL INFORMATION

### Data Deposition and Access

The interpreted fusion variants have been deposited in ClinVar (https://www.ncbi.nlm.nih.gov/clinvar/) under accession numbers SCV000845744 and SCV000845775. The aligned sequence data set for Case 2 has been deposited at the Genomic Data Commons (study ID: 29125) under accession number phs001628. The GDC number for this patient is A2812D. The RNA-seq data set for Case 1 will be submitted to GDC. The data is available upon request from the corresponding author.

### Ethics Statement

The study was approved by the Institutional Review Board (IRB) at Oregon Health and Science University. Samples were obtained with written, informed consent from all patients.

### Acknowledgments

The authors thank Beth Wilmot, Daniel Bottomly, and Shannon K. McWeeney for the RNA-seq fusion analysis. The authors thank Kara Johnson for general help. J.W.T. was supported by The Leukemia & Lymphoma Society, the V Foundation for Cancer Research, the Gabrielle's Angel Foundation for Cancer Research, and the National Cancer Institute (1R01CA183947-01, 1U01CA217862, 1U54CA224019, 1U01CA214116). H.Z. received the Medical Research Foundation (MRF) grant and Collins Trust Award.

### Author Contributions

H.Z. performed PCR validation, created the figure, and wrote the manuscript. A.P. and E.H. contributed to experimental design and data analysis. N.L. and K.-H.T.D. provided clinical information on the patients. S.M. and S.O. performed cytogenetic and FISH analysis. J.W.T. guided experimental design, data analysis, and manuscript revisions. K.-H.T.D., A.P., and S.M. performed a critical review of the manuscript.

### Competing Interest Statement

Research support for J.W.T. is received from Aptose, Array, AstraZeneca, Constellation, Genentech, Gilead, Incyte, Janssen, Seattle Genetics, Syros, Takeda, and the Scientific Advisory Board for Leap Oncology. All other authors declare no conflict of interest.

### Referees

Courtney DiNardo

Anonymous

## Supplementary Material

Supplemental Material

## References

[MCS003079ZHAC1] Arsham MS, Shaffer LG. 2017 ISCN: the universal language of cytogenetics. In The AGT cytogenetics laboratory manual (ed. Arsham MS, ), pp. 359–428. Wiley-Blackwell, New York.

[MCS003079ZHAC2] Chonabayashi K, Hishizawa M, Matsui M, Kondo T, Ohno T, Ishikawa T, Takaori-Kondo A. 2014 Successful allogeneic stem cell transplantation with long-term remission of *ETV6*/*FLT3*-positive myeloid/lymphoid neoplasm with eosinophilia. Ann Hematol 93: 535–537. 23873282 10.1007/s00277-013-1843-9

[MCS003079ZHAC3] Chung A, Hou Y, Ohgami RS, Von Gehr A, Fisk DG, Roskin KM, Li X, Gojenola L, Bangs CD, Arber DA, 2017 A novel *TRIP11*-*FLT3* fusion in a patient with a myeloid/lymphoid neoplasm with eosinophilia. Cancer Genet 216–217: 10–15. 10.1016/j.cancergen.2017.05.001 PMC12396770 29025582

[MCS003079ZHAC4] Falchi L, Mehrotra M, Newberry KJ, Lyle LM, Lu G, Patel KP, Luthra R, Popat U, Verstovsek S. 2014 *ETV6*-*FLT3* fusion gene-positive, eosinophilia-associated myeloproliferative neoplasm successfully treated with sorafenib and allogeneic stem cell transplant. Leukemia 28: 2090–2092. 24854988 10.1038/leu.2014.168 PMC4824944

[MCS003079ZHAC5] Grand FH, Iqbal S, Zhang L, Russell NH, Chase A, Cross NCP. 2007 A constitutively active *SPTBN1*-*FLT3* fusion in atypical chronic myeloid leukemia is sensitive to tyrosine kinase inhibitors and immunotherapy. Exp Hematol 35: 1723–1727. 17764812 10.1016/j.exphem.2007.07.002

[MCS003079ZHAC6] Hosseini N, Craddock KJ, Salehi-Rad S, Brennan S, Bailey DJ, Brandwein JM, Porwit A. 2014 *ETV6*/*FLT3* fusion in a mixed-phenotype acute leukemia arising in lymph nodes in a patient with myeloproliferative neoplasm with eosinophilia. J Hematop 7: 71–77.

[MCS003079ZHAC7] Kim D, Salzberg SL. 2011 TopHat-Fusion: an algorithm for discovery of novel fusion transcripts. Genome Biol 12: R72. 21835007 10.1186/gb-2011-12-8-r72 PMC3245612

[MCS003079ZHAC8] Krause DS, Van Etten RA. 2005 Tyrosine kinases as targets for cancer therapy. N Engl J Med 353: 172–187. 16014887 10.1056/NEJMra044389

[MCS003079ZHAC9] Sheng G, Zeng Z, Pan J, Kou L, Wang Q, Yao H, Wen L, Ma L, Wu D, Qiu H, 2017 Multiple *MYO18A*-*PDGFRB* fusion transcripts in a myeloproliferative neoplasm patient with t(5;17)(q32;q11). Mol Cytogenet 10: 4. 28261327 10.1186/s13039-017-0306-8 PMC5329908

[MCS003079ZHAC10] Stirewalt DL, Radich JP. 2003 The role of FLT3 in haematopoietic malignancies. Nat Rev Cancer 3: 650–665. 12951584 10.1038/nrc1169

[MCS003079ZHAC11] Troadec E, Dobbelstein S, Bertrand P, Faumont N, Trimoreau F, Touati M, Chauzeix J, Petit B, Bordessoule D, Feuillard J, 2017 A novel t(3;13)(q13;q12) translocation fusing *FLT3* with *GOLGB1*: toward myeloid/lymphoid neoplasms with eosinophilia and rearrangement of *FLT3*? Leukemia 31: 514–517. 27795560 10.1038/leu.2016.304 PMC5292680

[MCS003079ZHAC12] Tyner JW, Yang WF, Bankhead A 3rd, Fan G, Fletcher LB, Bryant J, Glover JM, Chang BH, Spurgeon SE, Fleming WH, 2013 Kinase pathway dependence in primary human leukemias determined by rapid inhibitor screening. Cancer Res 73: 285–296. 23087056 10.1158/0008-5472.CAN-12-1906 PMC3537897

[MCS003079ZHAC13] Tzankov A, Sotlar K, Muhlematter D, Theocharides A, Went P, Jotterand M, Horny H-P, Dirnhofer S. 2008 Systemic mastocytosis with associated myeloproliferative disease and precursor B lymphoblastic leukaemia with t(13;13)(q12;q22) involving *FLT3*. J Clin Pathol 61: 958–961. 18663058 10.1136/jcp.2008.058073

[MCS003079ZHAC14] Ussowicz M, Jaśkowiec A, Meyer C, Marschalek R, Chybicka A, Szczepański T, Haus O. 2012 A three-way translocation of MLL, MLLT11, and the novel reciprocal partner gene *MYO18A* in a child with acute myeloid leukemia. Cancer Genet 205: 261–265. 22682626 10.1016/j.cancergen.2012.02.006

[MCS003079ZHAC15] Vu HA, Xinh PT, Masuda M, Motoji T, Toyoda A, Sakaki Y, Tokunaga K, Sato Y. 2006 *FLT3* is fused to *ETV6* in a myeloproliferative disorder with hypereosinophilia and a t(12;13)(p13;q12) translocation. Leukemia 20: 1414–1421. 16761019 10.1038/sj.leu.2404266

[MCS003079ZHAC16] Walz C, Chase A, Schoch C, Weisser A, Schlegel F, Hochhaus A, Fuchs R, Schmitt-Gräff A, Hehlmann R, Cross NC, 2005 The t(8;17) (p11;q23) in the 8p11 myeloproliferative syndrome fuses *MYO18A* to *FGFR1*. Leukemia 19: 1005–1009. 15800673 10.1038/sj.leu.2403712

[MCS003079ZHAC17] Walz C, Erben P, Ritter M, Bloor A, Metzgeroth G, Telford N, Haferlach C, Haferlach T, Gesk S, Score J, 2011 Response of *ETV6*-*FLT3*-positive myeloid/lymphoid neoplasm with eosinophilia to inhibitors of FMS-like tyrosine kinase 3. Blood 118: 2239–2242. 21705501 10.1182/blood-2011-03-343426

[MCS003079ZHAC18] Wandt H, Haferlach T, Thiede C, Ehninger G. 2010 WHO classification of myeloid neoplasms and leukemia. Blood 115: 748–749. 20093414 10.1182/blood-2009-11-248302 PMC2810993

[MCS003079ZHAC19] Zhang H, Reister Schultz A, Luty S, Rofelty A, Su Y, Means S, Bottomly D, Wilmot B, McWeeney SK, Tyner JW. 2017 Characterization of the leukemogenic potential of distal cytoplasmic *CSF3R* truncation and missense mutations. Leukemia 31: 2752–2760. 28439110 10.1038/leu.2017.126 PMC5682244

