# Supplementary material for: Two myeloid leukemia cases with rare FLT3 fusions
Source: Cold Spring Harb Mol Case Stud. 2018 Dec;4(6):a003079. doi: 10.1101/mcs.a003079 (PMC6318770; doi:10.1101/mcs.a003079)
Supplement: Supplemental Material [file supp_4_6_a003079__index.html]

Supplemental Material 

# Two myeloid leukemia cases with rare *FLT3* fusions

## Supplemental Material

- Supplemental\_Data.docx
- Supplemental\_Table\_2.xlsx
