# Supplementary material for: Two myeloid leukemia cases with rare FLT3 fusions
Source: Cold Spring Harb Mol Case Stud. 2018 Dec;4(6):a003079. doi: 10.1101/mcs.a003079 (PMC6318770; doi:10.1101/mcs.a003079)
Supplement: Supplemental Material [file supp_mcs.a003079_Supplemental_Data.docx]

**Supplementary data**

**Supplementary Table 1: GeneTrails AML/MDS Gene Panel**

| ASXL1 | HRAS | NPM1 |
| --- | --- | --- |
| BCOR | IDH1 | NRAS |
| CBL | IDH2 | PAX5 |
| CBL-B | PTPN11 | SF3B1 |
| CEBPA | RUNX1 | SRSF2 |
| CSF3R | JAK2 | STAT3 |
| DNMT3A | JAK3 | TET2 |
| ETV6 | KDM6A/UTX | TP53 |
| EZH2 | KIT | U2AF35(U2AF1) |
| FLT3 | KRAS | WT1 |
| GATA1 | MLL | ZRSR2 |
| GATA2 | MPL |  |

More information please find here: <https://knightdxlabs.ohsu.edu/>

**Supplementary Table 3. Mutations detected in top 10 Sorafenib sensitive specimens**

| Specimen ID | IC50 (uM) | Common Driver mutation |  |
| --- | --- | --- | --- |
| 13-00058 | 0.608538 | NRAS, NPM1, U2AF1, ASXL1 |  |
| 14-00512 | 0.600852 | CEBP1, STAT3, RUNX1, ASXL1, EZH2, SRSF2, TET2 |  |
| 13-00102 | 0.266064 | NA |  |
| 13-00093 | 0.171143 | NA |  |
| 13-00656 | 0.161458 | MYO18A-FLT3 fusion |  |
| 15-00830 | 0.156605 | NRAS, ASXL1 |  |
| 14-00079 | 0.12943 | ETV6-FLT3 fusion |  |
| 14-00126 | 0.087811 | ETV6-FLT3 fusion |  |
| 13-00176 | 0.057658 | NA |  |
| 13-00556 | 0.045187 | NA |  |

NA: not available.

**Supplementary Table 4. Mutations detected in top 10 Quizartinib sensitive specimens**

| Specimen ID | IC50 (uM) | Common Driver mutation | |  |
| --- | --- | --- | --- | --- |
| 12-00331 | 0.054921 | NA |  |  |
| 15-00949 | 0.049477 | NA |  |  |
| 15-00138 | 0.049425 | NA |  |  |
| 14-00126 | 0.028711 | ETV6-FLT3 fusioin |  |  |
| 13-00241 | 0.027972 | CBL, TET2, EP300, SRSF2, ASXL1 | |  |
| 14-00461 | 0.027485 | NA |  |  |
| 13-00139 | 0.025912 | NA |  |  |
| 14-00079 | 0.021897 | ETV6-FLT3 fusion |  |  |
| 15-00557 | 0.01178 | MYO18A-FLT3 fusion |  |  |
| 13-00139 | 0.011163 | JAK2, SF3B1, TET2, ASXL1 | |  |

NA: not available.

**Supplementary Table 5. Mutations detected in top 10 Imatinib sensitive specimens**

| Specimen ID | IC50 (uM) | Common Driver mutation | |
| --- | --- | --- | --- |
| 13-00556 | 0.75201 | NA |  |
| 08-00179 | 0.672022 | NA |  |
| 13-00246 | 0.635024 | PTPN11, ASXL1, SRSF2 | |
| 12-00307 | 0.631767 | NA |  |
| 13-00093 | 0.508395 | NA |  |
| 13-00102 | 0.488034 | NA |  |
| 14-00475 | 0.308214 | CSF3R, ASXL1 |  |
| 11-00188 | 0.276257 | NA |  |
| 13-00101 | 0.232602 | NA |  |
| 14-00457 | 0.208722 | NA |  |

NA: not available.

**References:**

Tyner, J.W., Yang, W.F., Bankhead, A., Fan, G., Fletcher, L.B., Bryant, J., Glover, J.M., Chang, B.H., Spurgeon, S.E., Fleming, W.H., et al. (2013). Kinase pathway dependence in primary human leukemias determined by rapid inhibitor screening. Cancer Res. *73*, 285–296.

Zhang, H., Reister Schultz, A., Luty, S., Rofelty, A., Su, Y., Means, S., Bottomly, D., Wilmot, B., McWeeney, S.K., and Tyner, J.W. (2017). Characterization of the leukemogenic potential of distal cytoplasmic CSF3R truncation and missense mutations. Leukemia.
